# Urinary Phthalate Metabolites Are Associated with Body Mass Index and Waist Circumference in Chinese School Children

**DOI:** 10.1371/journal.pone.0056800

**Published:** 2013-02-20

**Authors:** Hexing Wang, Ying Zhou, Chuanxi Tang, Yanhong He, Jingui Wu, Yue Chen, Qingwu Jiang

**Affiliations:** 1 Key Laboratory of Public Health Safety of Ministry of Education, School of Public Health, Fudan University, Shanghai, China; 2 Centers for Disease Control and Prevention of Changning District, Shanghai, China; 3 Department of Laboratory Medicine, Anting Hospital, Shanghai, China; 4 Department of Epidemiology and Community Medicine, Faculty of Medicine, University of Ottawa, Ottawa, Ontario, Canada; Stony Brook University, Graduate Program in Public Health, United States of America

## Abstract

**Background:**

Lab studies have suggested that ubiquitous phthalate exposures are related to obesity, but relevant epidemiological studies are scarce, especially for children.

**Objective:**

To investigate the association of phthalate exposures with body mass index (BMI) and waist circumference (WC) in Chinese school children.

**Methods:**

A cross-sectional study was conducted in three primary and three middle schools randomly selected from Changning District of Shanghai City of China in 2011–2012. According to the physical examination data in October, 2011, 124 normal weight, 53 overweight, and 82 obese students 8–15 years of age were randomly chosen from these schools on the basis of BMI-based age- and sex-specific criterion. First morning urine was collected in January, 2012, and fourteen urine phthalate metabolites (free plus conjugated) were determined by ultra-performance liquid chromatography coupled to tandem mass spectrometry. Multiple linear regression was used to explore the associations between naturally log-transformed urine phthalate metabolites and BMI or WC.

**Results:**

The urine specific gravity-corrected concentrations of nine urine phthalate metabolites and five molar sums were positively associated with BMI or WC in Chinese school children after adjustment for age and sex. However, when other urine phthalate metabolites were included in the models together with age and sex as covariables, most of these significant associations disappeared except for mono (2-ethylhexyl) phthalate (MEHP) and monoethyl phthalate (MEP). Additionally, some associations showed sex- or age-specific differences.

**Conclusions:**

Some phthalate exposures were associated with BMI or WC in Chinese school children. Given the cross-sectional nature of this study and lack of some important obesity-related covariables, further studies are needed to confirm the associations.

## Introduction

Phthalates, the diesters of phthalic acid, are extensively used as plasticizers and solvents in industrial, medical, and consumer products [1]. Applications of phthalates depend on their chemical structures, by which they are classified into two groups of high and low molecular weight [2]. High molecular weight phthalates, such as di(2-ethylhexyl) phthalate (DEHP), butyl benzyl phthalate (BBzP), di-iso-nonyl phthalate (DiNP), di-iso-decyl phthalate (DiDP) and dioctyl phthalate (DOP), are primarily used in the production of polyvinylchloride plastics (PVC) [3], [4]. Low molecular weight phthalates, such as dimethyl phthalate (DMP), diethyl phthalate (DEP), di-n-butyl phthalate (DBP), and di-iso-butyl phthalate (DiBP), are often used in making personal care products (e.g. perfumes, lotions and cosmetics), varnishes, and coatings [5], [6]. Humans can be exposed to phthalates through ingestion, inhalation, and dermal contact as well as during intrauterine development through placenta [7]. As a result of ubiquitous use of these phthalate-related products, human exposures to phthalates have been considered to be extensive [8], [9]. Experimental studies have demonstrated that some phthalates are endocrine disrupting chemicals (EDCs), and have reproductive or developmental toxicity [10].

There is increasing evidences suggesting that some EDCs can act as obesogens and interfere with body’s natural weight control mechanisms by disrupting adipose tissue biology, endocrine hormone systems, or central hypothalamic-pituitary-adrenal axis [11], [12]. For this, the National Toxicology Program (NTP) organized a special workshop to discuss the phthalates as potential obesogen and recommended subsequent research strategy in 2011 [13]. Lab and population studies have suggested that phthalate exposures can promote adipogenesis by diverse biologic mechanisms, such as interfering with the functions of steroid hormones or thyroid hormones, and the inappropriate activation of peroxisome proliferator-activated receptors (PPARs) [14]–[17].

Sex hormones are critical to the regulation of body composition, fuel homeostasis, and reproduction in humans [18]. Some lab studies have observed that DEHP and (mono(2-ethylhexyl) phthalate (MEHP) decrease the biosynthesis of steroid hormones, such as estradiol and testosterone, by suppressing aromatase transcript levels [19], [20]. Some phthalates, such as DiBP, DBP, dicyclohexyl phthalate (DCHP) and DEHP, were found to activate human estrogen receptors, human androgen receptors, and glucocorticoid receptors [21]. Thyroid function is critical to the maintenance of basal metabolism. Shen et al. found that DBP and MBP altered expression of selected thyroid hormone response genes by Xenopus laevis [22]. Epidemiological studies also observed an inverse association between urinary phthalate metabolites, such as monoethyl phthalate (MEP), mono-n-butyl phthalate (MBP), monobenzyl phthalate (MBzP), and MEHP, and thyroid hormone serum levels in children and in men [23], [24]. Kitahara et al. observed that levels of serum TSH and free T3 were positively associated with body mass index (BMI) and waist circumference (WC) among euthyroid adults [25]. PPARs play a major role in adipocyte differentiation and energy storage. Bility et al. observed that several phthalate metabolites, such as MBzP, monoisononyl phthalate (MiNP), monooctyl phthalate (MOP) and MEHP, could activate mouse and human PPAR-α, PPAR-β, and PPAR-γ although the ability of these metabolites to activate these receptors considerably varied [26]. Feige et al. reported that MEHP was a selective PPAR-γ modulator that promotes adipogenesis [27].

However, epidemiological studies that investigate the associations of postnatal phthalate exposures with obesity or body size measurements are scarce. To our knowledge, there were four epidemiological studies for this, including two cross-sectional studies and one longitudinal study involved in children, another cross-sectional one in men [24], [28]–[30]. Among these studies, phthalate metabolites, such as MEP, MiBP, MBP, MBzP, and some metabolites of DEHP, were found to be significantly associated with BMI or WC.

Children and adolescents are in rapid growth and development and are more susceptible to external disturbance than adults. Moreover, it has been reported that children are extensively exposed to phthalates and the exposure levels are higher compared with adults [31], [32]. Body mass index is a reasonable measure of fatness in children and adolescents and used as an indicator of overall obesity, while WC could reflect the accumulation of abdominal fat tissue and is used as an indicator of central obesity [33], [34]. In this study we examined the association of urine phthalate metabolite concentrations with BMI and WC in Chinese school children.

## Materials and Methods

### Ethics Statement

The study was approved by the Ethical Review Board of the School of Public Health of Fudan University. Guardians provided written informed consent before the study.

### Study Population

The study design and study population has been described in detail in a previous report of the association of urine bisphenol a (BPA) with BMI [35]. Briefly, three primary schools and three middle schools were randomly selected from 26 primary and 30 middle candidate schools in Changning District of Shanghai City in China. From each selected school, 20 obese, 10 overweight, and 30 normal weight students aged 8–15 years were randomly chosen in the basis of the most recent yearly regular physical examination conducted in October, 2011. A total of 360 eligible students were selected and they were all Han people. Of these 360 subjects, 53 did not provide urine samples and 48 provided urine samples but not first morning urine. Therefore, 259 participants were available for the current analysis. The distributions of participants by age, sex, and BMI subgroups (normal weight, overweight, and obesity) were listed in Table 1.

**Table 1:** Arithmetic mean (standard deviation) of waist circumference (WC) and body mass index (BMI) in 259 school children of Shanghai, China in 2011–2012.

	No. (%)	WC (cm)	p-Value	BMI (kg/m^2^)	p-Value
All	259 (100.0)	72.1±12.6		21.3±4.6	
By age (years)					
8–9	64 (24.7)	64.5±.9.9	<0.001^a^	18.7±4.0	<0.001^a^
10–11	80 (30.9)	69.4±11.1		20.5±3.7	
12–13	75 (29.0)	76.9±12.1		22.3±4.4	
14–15	40 (15.4)	80.1±12.2		25.0±4.6	
By sex					
Female	129 (49.8)	70.7±10.7	0.302^b^	21.1±4.3	0.664^a^
Male	130 (50.2)	73.4±14.1		21.4±4.9	
By BMI					
Normal weight	124 (47.9)	63.4±8.1	<0.001^a^	17.4±2.1	<0.001^c^
Overweight	53 (20.4)	75.2±8.7		23.0±1.9	
Obesity	82 (31.7)	82.7±11.1		26.0±3.2	

a Analysis of variance,

b Mann-Whitney rank test instead of analysis of variance due to heterogeneity of variance.

c Kruskai-Wallis rank test instead of analysis of variance due to heterogeneity of variance.

### Anthropometric Measurements

Body weight (kg) and height (cm) were measured using the same type of apparatus and followed the standard procedures recommended by Cameron [36]. Each technician was required to pass a training course for the anthropometric measurements. Subjects were required to wear light clothes and stand straight and barefoot when being measured. Waist circumference (cm) was measured midway between the lowest rib and the superior border of the iliac crest with an inelastic measuring tape at the end of normal expiration to the nearest 0.1 cm [34]. Body mass index was calculated as weight in kilograms divided by height in meters squared. Normal weight, overweight, and obese participants in this study were identified according to BMI-based age- and sex-specific criterion proposed by the Working Group on Obesity in China (WGOC). This criterion was based on a large representative Chinese population 7–18 years of age and the specific BMI cut-off values were listed in Table S1 in Supporting Information File S1 [37].

### Measurement of Urine Phthalate Metabolites

For a better comparability of urine phthalate metabolites among individuals, first morning urine was collected and participants themselves carried out the urine collection. In order to minimize the contamination of phthalates during urine sampling and storing, urine samples were collected in glass centrifuge tubes, which was rinsed by acetone and baked at 350°C for 2 hours, and the participants were told to avoid a contact of urine with plastic products in the process of urine collection. The collected urine samples were transported to lab as soon as possible and stored in dark at −20°C until analysis. All the urine samplings were collected between January 3 and 6, 2012.

Fourteen phthalate metabolites studied here included MEHP, mono(2-ethyl-5-oxohexyl) phthalate (MEOHP), mono(2-ethyl-5-carboxypentyl) phthalate (MECPP), mono(2-ethyl-5-hydroxyhexyl) phthalate (MEHHP), MBP, monoisobutyl phthalate (MiBP), MEP, mono[(2-carboxymethyl) hexyl] phthalate (MCMHP), mono(4-hydroxybutyl) phthalate (MHBP), monomethyl phthalate (MMP), monocyclohexyl phthalate (MCHP), MBzP, monoisononyl phthalate (MiNP), and monooctyl phthalate (MOP). MEHP is the primary metabolite of DEHP, and MEHHP, MECPP, MCMHP, and MEOHP are secondary metabolites of DEHP. MBP and MHBP are primary and secondary metabolites of DBP, respectively. MiBP is derived from DiBP, MMP from DMP, MEP from DEP, MCHP from DCHP, MBzP from BBzP, MiNP from DiNP, and MOP from DOP.

Fourteen urine phthalate metabolites (free and conjugated) were analyzed using a method modified from Guo et al. [1]. Briefly, after an aliquot (1.0 mL) of urine sample was enzymatically hydrolyzed and purified by solid-phase extraction, the phthalate metabolites in urine extract were resolved by reversed-phase ultra-performance liquid chromatography, detected by electrospray ionization tandem mass spectrometry, and quantified by an isotope internal standard curve method.

All urine analyses were carried out in random and information on BMI subgroup was blinded to analytical technicians. To minimize a potential systematic drift caused by analytical process, all the analyses of urine samples were performed in a short time span (all the analyses of urine samples were completed in May 2012) and by the same analytical team. The recoveries of phthalate metabolites of spiked urine samples used for quality control in the process of whole urine analyses varied between 70.3% and 124%, and their standard deviations varied between 9.2% and 19.1%. The detailed analytical method was supplied in Supporting Information File S1 (Tables S2 and S3 in Supporting Information File S1).

### Statistical Analyses

For measurements below the limit of detection (LOD, see supplementary information File S1), a default value of LOD divided by the square root of 2 was assigned, which is a method of handling nondetectable values that produces reasonably nonbiased means and standard deviations [38]. In addition to twelve individual phthalate metabolites, we combined some phthalate metabolites into molar sums based on their molecular-weight or parent phthalates. Sum of low molecular-weight phthalate metabolites (MWP) comprised of MBP, MHBP, MiBP, MMP, and MEP, and sum of high MWP comprised of five metabolites of DEHP (MEHP, MECPP, MEHHP, MEOHP, MCMHP), MCHP, and MBzP. By respective molar sum of each metabolites of constructed sums, sum of metabolites of DBP (MBP and MHBP) and sum of low MWP were converted to MBP (molecular weight 222) mass concentrations (ng/mL), and sum of metabolites of DEHP (MEHP, MEHHP, MECPP, MCMHP, and MEOHP), sum of high MWP, and sum of all phthalate metabolites were converted to MEHP (molecular weight 278) mass concentrations (ng/mL). These sums have the same unit as phthalate metabolites.

Due to the glucuronidation of phthalate metabolites in the liver and its elimination by active tubular secretion, creatinine correction may not be appropriate for urine phthalate metabolite concentration [8], [39]. Additionally, BMI could predict the urine creatinine concentration, and therefore, the associations between phthalate metabolites and BMI may be altered by creatinine adjustment [40]. For this reason, we used specific gravity to correct for urinary dilution, as recommended by Hauser et al. [39]. Specific gravity was measured using a handheld refractometer (Atago PAL 10-S, Tokyo, Japan). The correction formula was P_c_ = P × [(1.024–1)/(SG –1)], where P_c_ is the specific gravity-corrected phthalate metabolite concentration (ng/mL), P is the experimental phthalate metabolite concentration (ng/mL), and SG is the specific gravity of the urine sample [41].

For descriptive statistics, arithmetic mean and standard deviation (SD) of BMI and WC, and geometric mean (GM), 95% confidence interval (CI), and median of SG-uncorrected urine phthalate metabolite concentrations were calculated for all subjects or by sex, age, and BMI subgroups (normal weight, overweight, and obesity) (Table 1). Because the urinary concentration values of phthalate metabolite were not normally distributed, a natural log-transformation was applied to normalize the data. Analysis of variance was used to investigate the association of SG-uncorrected urine phthalate metabolite concentrations with age (8–11 and 12–15 age groups), sex, and BMI subgroups (normal weight, overweight, and obesity) in all subjects (Tables 2 and 3). When heterogeneity of variance was encountered, rank test was used instead of analysis of variance.

**Table 2:** Geometric mean (median), ng/mL) of urine phthalate metabolite concentrations before urine specific gravity correction associated with age and sex in 259 school children of Shanghai, China in 2011–2012.

Compounds	DF^a^ (%)	All	Age (year)			Sex		
			8–11	12–15	p-Value^b^	Male	Female	p-Value^c^
MEHP	100	21.3±1.0(21.1)	18.4±1.1(18.6)	25.5±1.1(24.4)	<0.001	20.5±1.1(21.6)	22.0±1.1(21.0)	0.224
MECPP	100	28.8±1.1(29.8)	27.9±1.1(28.6)	30.0±1.1(32.4)	0.397	27.1±1.1(32.2)	30.6±1.1(26.2)	0.146
MEHHP	100	16.1±1.0(15.7)	16.0±1.1(15.5)	16.0±1.1(16.2)	0.884	15.2±1.1(16.7)	16.9±1.1(14.8)	0.171
MEOHP	100	22.9±1.1(22.9)	22.2±1.1(22.4)	23.8±1.1(23.7)	0.475	21.3±1.1(27.5)	24.8±1.1(21.1)	0.127
MCMHP	99.6	22.0±1.1(22.9)	20.1±1.1(19.7)	25.0±1.1(27.9)	0.058	20.5±1.1(26.3)	23.6±1.1(20.0)	0.220
Sum of DEHP	100	117.3±1.0(122.5)	109.2±1.1(117.7)	129.2±1.1(135.6)	0.048	111.7±1.1(128.1)	124.5±1.1(108.1)	0.163
MCHP	94.6	0.8±1.1(0.9)	0.6±1.1(0.7)	1.2±1.1(1.3)	<0.001	0.7±1.1(1.0)	0.9±1.1(0.9)	0.226
MBzP	38.6	0.068±1.08(0.05)	0.069±1.11(0.05)	0.066±1.13(0.05)	0.572^d^	0.068±1.12(0.05)	0.068±1.13(0.05)	0.841^d^
Sum of high MWP	100	119.1±1.0(124.8)	109.9±1.1(119.0)	131.6±1.1(135.2)	0.001	113.3±1.1(111.7)	126.5±1.1(129.6)	0.224
MBP	99.6	47.5±1.1(47.2)	42.9±1.1(40.7)	53.0±1.1(53.7)	0.076	44.7±1.1(52.4)	50.4±1.1(43.1)	0.219
MHBP	99.6	26.6±1.1(28.2)	23.6±1.1(22.8)	31.2±1.1(35.5)	0.050	24.0±1.1(29.6)	29.4±1.1(26.3)	0.187
Sum of DBP	100	84.8±1.1(81.7)	77.5±1.1(75.2)	95.6±1.1(99.9)	0.042	90.0±1.1(87.6)	79.8±1.1(77.1)	0.221
MiBP	100	38.9±1.1(37.4)	37.7±1.18(35.1)	40.0±1.1(38.4)	0.578	35.5±1.1(42.4)	42.5±1.1(34.3)	0.087
MMP	100	9.6±1.1(9.1)	7.9±1.1(8.0)	12.3±1.1(13.4)	<0.001	9.4±1.1(9.4)	9.8±1.1(9.1)	0.646
MEP	98.9	15.3±1.1(15.9)	11.6±1.1(10.8)	22.0±1.1(21.0)	<0.001	15.6±1.1(16.7)	15.0±1.1(14.8)	0.928
Sum of low MWP	100	175.9±1.1(177.8)	159.2±1.1(154.1)	198.3±1.1(206.7)	0.459	183.1±1.1(183.0)	167.3±1.1(161.4)	0.206
Sum of all	100	399.4±1.0(406.8)	365±1.1(367.9)	445.9±1.1(465.3)	0.092	407.5±1.1(416.6)	387.6±1.1(379.0)	0.536

a Detection frequency.

b Analysis of variance adjusted for sex.

c Analysis of variance adjusted for age.

d Mann-Whitney rank test instead of analysis of variance due to abnormal distribution.

**Table 3:** Geometric mean (median), ng/mL) of urine phthalate metabolite concentrations before specific gravity correction associated with excess body weight after adjustment for sex and age in 259 school children of Shanghai, China in 2011–2012.

Compounds	Normal weight	Over weight	Obesity	p-Value
MEHP	18.5±1.1(18.0)	21.3±1.1(22.9)	26.0±1.1(24.0)	0.006
MECPP	27.7±1.1(28.4)	23.1±1.1(24.8)	35.2±1.1(35.2)	0.020
MEHHP	15.3±1.1(15.2)	12.8±1.1(13.8)	19.7±1.1(19.0)	0.011
MEOHP	21.3±1.1(22.3)	19.5±1.1(21.2)	28.2±1.1(30.2)	0.047
MCMHP	20.5±1.1(20.4)	17.5±1.2(17.8)	28.8±1.1(27.1)	0.015
Sum of DEHP	108.1±1.1(115.7)	105.4±1.1(106.0)	144.5±1.1(135.5)	0.019
MCHP	0.7±1.1(0.70)	0.8±1.2(0.93)	1.1±1.1(1.23)	0.060
Sum of high MWP	109.9±1.1(116.6)	105.6±1.1(108.6)	146.9±1.1(137.6)	0.006^a^
MBP	40.4±1.1(41.1)	48.4±1.2(45.7)	58.6±1.1(57.1)	0.161
MHBP	23.3±1.1(22.8)	27.4±1.2(28.0)	31.8±1.1(32.3)	0.084
Sum of DBP	75.2±1.1(67.3)	88.2±1.2(77.3)	101±1.1(98.3)	0.018^a^
MiBP	34.5±1.1(31.1)	35.5±1.1(37.2)	49.4±1.1(50.6)	0.009
MMP	8.7±1.1(8.9)	10.0±1.2(8.9)	11.0±1.1(9.7)	0.310
MEP	11.0±1.1(11.9)	19.7±1.2(13.8)	21.5±1.1(21.0)	0.009
Sum of low MWP	148.4±1.1(144.7)	194.4±1.1(200.8)	210.6±1.1(210.4)	0.003^a^
Sum of all	343.8±1.1(362.4)	403.4±1.1(423.0)	492.7±1.1(500.1)	0.004

a Kruskai-Wallis rank test instead of analysis of variance due to heterogeneity of variance.

Multiple linear regres­sion analysis was also performed to study the association of SG-corrected urine phthalate metabolite concentrations with BMI and WC values (Tables 4 and 5). Linear regression analysis included only phthalate metabolites detectable in more than 50% of children. Because a fixed proportion of BMI subgroups (30 normal weight, 10 overweight, and 20 obese children) was used to select participants from each school with different selection probabilities for the BMI subgroups, it might generate a bias when estimating the associations in combined population of three BMI subgroups. We used sampling weights to calculate weighted parameter estimates in multiple linear regres­sion analysis [42], [43]. Because urinary phthalate metabolite concentrations were correlated with each other, especially between metabolites derived from one parent phthalate (see Table S4 in Supporting Information File S1), urine phthalate metabolites were first examined separately in crude analysis and adjusted model 1 with age and sex included as covariables, and then, in adjusted model 2, urine phthalate metabolites were also included as covariables besides age and sex. In addition to the regression models including all the subjects, stratified modeling by age (8–11 and 12–15 age groups) and sex was also conducted. The regression coefficient (β) represented the change of naturally log-transformed BMI or WC value per one unit of naturally log-transformed urine phthalate metabolite concentration. Analysis of variance was used to test for the interactions of urine phthalate metabolites with age subgroups (8–11 and 12–15 years of age) or sex by modeling them as main effects and interaction terms of naturally log-transformed BMI.

**Table 4:** Log-transformed urinary phthalate metabolite concentrations corrected by specific gravity (SG) associated with body mass index (BMI) in 259 school children of Shanghai, China in 2011–2012.

Compounds	Crude analysis^a^		Adjusted model 1^b^		Adjusted model 2	
	β^c^ (95% CI)	p-Value	β (95% CI)	p-Value	β (95% CI)	p-Value
MEHP	0.081 (0.044,0.118)	<0.001	0.058 (0.024,0.092)	0.001	0.048 (0.007,0.089)	0.021^d^
MECPP	0.018 (−0.014,0.050)	0.268	0.021 (−0.007,0.049)	0.149	−0.006 (−0.042,0.029)	0.724^d^
MEHHP	0.018 (−0.015,0.052)	0.280	0.026 (−0.004,0.055)	0.090	0.001 (−0.035,0.037)	0.955^d^
MEOHP	0.023 (−0.008,0.054)	0.139	0.025 (−0.002,0.052)	0.070	−0.001 (−0.037,0.036)	0.967^d^
MCMHP	0.033 (0.006,0.061)	0.018	0.026 (0.002,0.050)	0.036	0.009 (−0.024,0.041)	0.608^d^
Sum of DEHP	0.040 (0.006,0.075)	0.023	0.037 (0.006,0.067)	0.019	0.015 (−0.026,0.056)	0.469^d^
MCHP	0.052 (0.030,0.073)	<0.001	0.023 (0.002,0.044)	0.029	0.014 (−0.009,0.036)	0.233^e^
Sum of high MWP	0.042 (0.007,0.077)	0.020	0.039 (0.008,0.070)	0.014	0.021 (−0.020,0.062)	0.311^f^
MBP	0.028 (−0.003,0.059)	0.076	0.028 (0.001,0.055)	0.048	0.008 (−0.027,0.048)	0.654^g^
MHBP	0.030 (0.005,0.054)	0.018	0.023 (0.001,0.045)	0.044	0.008 (−0.020,0.036)	0.567^g^
Sum of DBP	0.047 (0.017,0.076)	0.002	0.035 (0.008,0.061)	0.010	0.030 (−0.008,0.068)	0.126^g^
MiBP	0.035 (0.012,0.059)	0.004	0.027 (0.006,0.048)	0.013	0.020 (−0.005,0.045)	0.120^g^
MMP	0.031 (0.003,0.060)	0.031	0.010 (−0.016,0.036)	0.437	−0.033 (−0.067,0.001)	0.055^h^
MEP	0.035 (0.018,0.052)	<0.001	0.025 (0.009,0.040)	0.002	0.022 (0.005,0.040)	0.011^i^
Sum of low MWP	0.057 (0.026,0.089)	<0.001	0.041 (0.012,0.069)	0.005	0.032 (−0.005,0.070)	0.088^j^
Sum of all	0.057 (0.024,0.091)	0.001	0.049 (0.020,0.079)	0.001	0.028 (−0.023,0.078)	0.285^k^

a Just included a variable of SG-corrected urine phthalate metabolites.

b Corrected for age and sex.

c β: Regression coefficient (log-transformed BMI/log-transformed metabolite concentration).

d Corrected for age, sex, sum of DBP, MCHP, MMP, and MEP.

e Corrected for age, sex, sum of DEHP, Sum of DBP, MMP, and MEP.

f Corrected for age, sex, sum of DBP, MMP, and MEP.

g Corrected for age, sex, sum of DEHP, MCHP, MMP, and MEP.

h Corrected for age, sex, sum of DEHP, MCHP, sum of DBP, and MEP.

i Corrected for age, sex, sum of DEHP, MCHP, sum of DBP, and MMP.

j Corrected for age, sex, sum of DEHP, and MCHP.

k Corrected for age, sex, sum of DEHP, and MEP.

**Table 5:** Log-transformed urinary phthalate metabolite concentrations corrected by specific gravity (SG) associated with waist circumference (WC) in 259 school children of Shanghai, China in 2011–2012.

	Crude analysis^a^		Adjusted model 1^b^		Adjusted model 2	
Compounds	β^c^ (95% CI)	p-Value	β (95% CI)	p-Value	β (95% CI)	p-Value
MEHP	0.063 (0.033,0.093)	<0.001	0.043 (0.016,0.070)	0.002	0.038 (0.006,0.071)	0.021^d^
MECPP	0.011(−0.015,0.036)	0.419	0.013 (−0.009,0.035)	0.249	−0.007 (−0.035,0.022)	0.642^d^
MEHHP	0.010 (−0.017,0.037)	0.455	0.017 (−0.007,0.040)	0.160	−0.002 (−0.030,0.027)	0.915^d^
MEOHP	0.017 (−0.008,0.041)	0.190	0.018 (−0.004,0.039)	0.103	0.001 (−0.028,0.030)	0.959^d^
MCMHP	0.023 (0.001,0.045)	0.042	0.017 (−0.002,0.036)	0.084	0.005 (−0.021,0.031)	0.706^d^
Sum of DEHP	0.029 (0.001,0.057)	0.044	0.026 (0.001,0.050)	0.038	0.012 (−0.021,0.044)	0.486^d^
MCHP	0.042 (0.025,0.059)	<0.001	0.019 (0.002,0.035)	0.025	0.013 (−0.005,0.031)	0.150^e^
Sum of high MWP	0.030 (0.001,0.058)	0.040	0.027 (0.003,0.052)	0.029	0.017 (−0.016,0.049)	0.310^f^
MBP	0.017 (−0.008,0.042)	0.191	0.015 (−0.007,0.037)	0.188	−0.003(−0.031,0.025)	0.849^g^
MHBP	0.015 (−0.006,0.036)	0.156	0.009 (−0.009,0.028)	0.319	−0.007 (−0.030,0.016)	0.550^g^
Sum of DBP	0.034 (0.010,0.058)	0.006	0.023 (0.002,0.044)	0.035	0.018 (−0.013,0.048)	0.256^g^
MiBP	0.030 (0.011,0.048)	0.002	0.022 (0.005,0.038)	0.011	0.019 (−0.001,0.038)	0.068^g^
MMP	0.022 (−0.001,0.045)	0.063	0.005 (−0.016,0.025)	0.662	−0.028 (−0.054, −0.001)	0.042^h^
MEP	0.028 (0.015,0.042)	<0.001	0.020 (0.008,0.032)	0.002	0.020 (0.006,0.033)	0.005^i^
Sum of low MWP	0.041 (0.016, 0.067)	0.002	0.027 (0.004,0.049)	0.022	0.019 (−0.011,0.048)	0.224^j^
Sum of all	0.041 (0.012,0.069)	0.005	0.031 (0.006,0.056)	0.016	−0.011 (−0.067,0.045)	0.701^k^

a Just included a variable of SG-corrected urine phthalate metabolites.

b Corrected for age and sex.

c β: Regression coefficient (log-transformed WC/log-transformed metabolite concentration).

d Corrected for age, sex, sum of DBP, MCHP, MMP, and MEP.

e Corrected for age, sex, sum of DEHP, Sum of DBP, MMP, and MEP.

f Corrected for age, sex, sum of DBP, MMP, and MEP.

g Corrected for age, sex, sum of DEHP, MCHP, MMP, and MEP.

h Corrected for age, sex, sum of DEHP, MCHP, sum of DBP, and MEP.

i Corrected for age, sex, sum of DEHP, MCHP, sum of DBP, and MMP.

j Corrected for age, sex, sum of DEHP, and MCHP.

k Corrected for age, sex, sum of DEHP, and MEP.

In adjusted model 2, in order to avoid the collinearity derived from strong correlations among daughter metabolites of one parent phthalates in linear regression model, only one daughter metabolite was included, whereas all others not. In this model, sum of DEHP was used to represent for five metabolites of DEHP, and sum of DBP was used to represent for MBP and MHBP. When one of the phthalate metabolites from DEHP or DBP was included in this model, sum of DBP or sum of DEHP was not added as covariable into the model. In addition, sum of high MWP, sum of low MWP, and sum of all were not used as independent covariables in all these models. The detail information about covariable for specific metabolites modelling was provided in Tables 4 and 5. Data analysis was performed using SPSS (version 17; SPSS, Inc., Chicago, IL, USA); p<0.05 was considered significant, and all statistical tests were two-sided.

## Results

Table 1 shows demographic characteristics and anthropometric measurements by age (8–9, 10–11, 12–13, and 14–15 years), sex and BMI subgroups (normal weight, overweight, and obesity). As expected, WC and/or BMI increased with age and BMI subgroups (p<0.001). There were no significant differences between males and females.

As shown in Table 2, phthalate exposure was prevalent. Except for MBzP, detection frequency of other eleven metabolites was 94.6% (MCHP) or above. Analysis of variance showed that four (MEHP, MCHP, MMP, and MEP) of twelve urine phthalate metabolites were significantly higher in children 12–15 years of age than those 8–11 years of age, and three (MCMHP, MBP, and MHBP) were marginally higher (p<0.10). No significant sex-related difference was observed for all urine phthalate metabolites. MOP and MiNP were not detected in all urine samples.

Table 3 shows the association of urinary metabolites with BMI grouped as normal weight, overweight, and obesity. Analysis of variance and Kruskai-Wallis rank test indicated that seven of eleven phthalate metabolites and five sums were significantly different among BMI groups. The average levels of MEHP, MiBP, MEP, sum of DBP, sum of low MW, and sum of all metabolites showed an increasing trend with BMI.

In multiple linear regression analysis, similar results were obtained before and after sampling weights were accounted for, and weighted estimates were presented. The detail sampling weights of each selected school was provided in Table S5 in Supporting Information File S1. Tables 4 and 5 showed that the regression results for the association of urine phthalate metabolites with BMI and WC, respectively. As shown in crude analysis, adjusted model 1, and adjusted model 2, the associations of phthalate metabolites with BMI or WC became weaker, and some association directions were even reversed after adjustment for more covariables.

Table 4 indicated that seven of eleven metabolites and five sums were positively associated with increasing BMI after adjustment for age and sex in adjusted model 1, but after additional adjustment for urine phthalate metabolites in adjusted model 2, only MEHP and MEP remained significant, and sum of low MWP showed a similar trend (p<0.1). As shown in Table 5, the significant associations of phthalate metabolites with WC decreased in the basis of those with BMI in crude analysis and adjusted model 1.and MEHP and MEP were still positively associated with WC in adjusted model 2, but compared to BMI, the association of MiBP with WC showed a positive trend (p<0.1) and MMP was inversely associated with WC.

**Figure 1:**
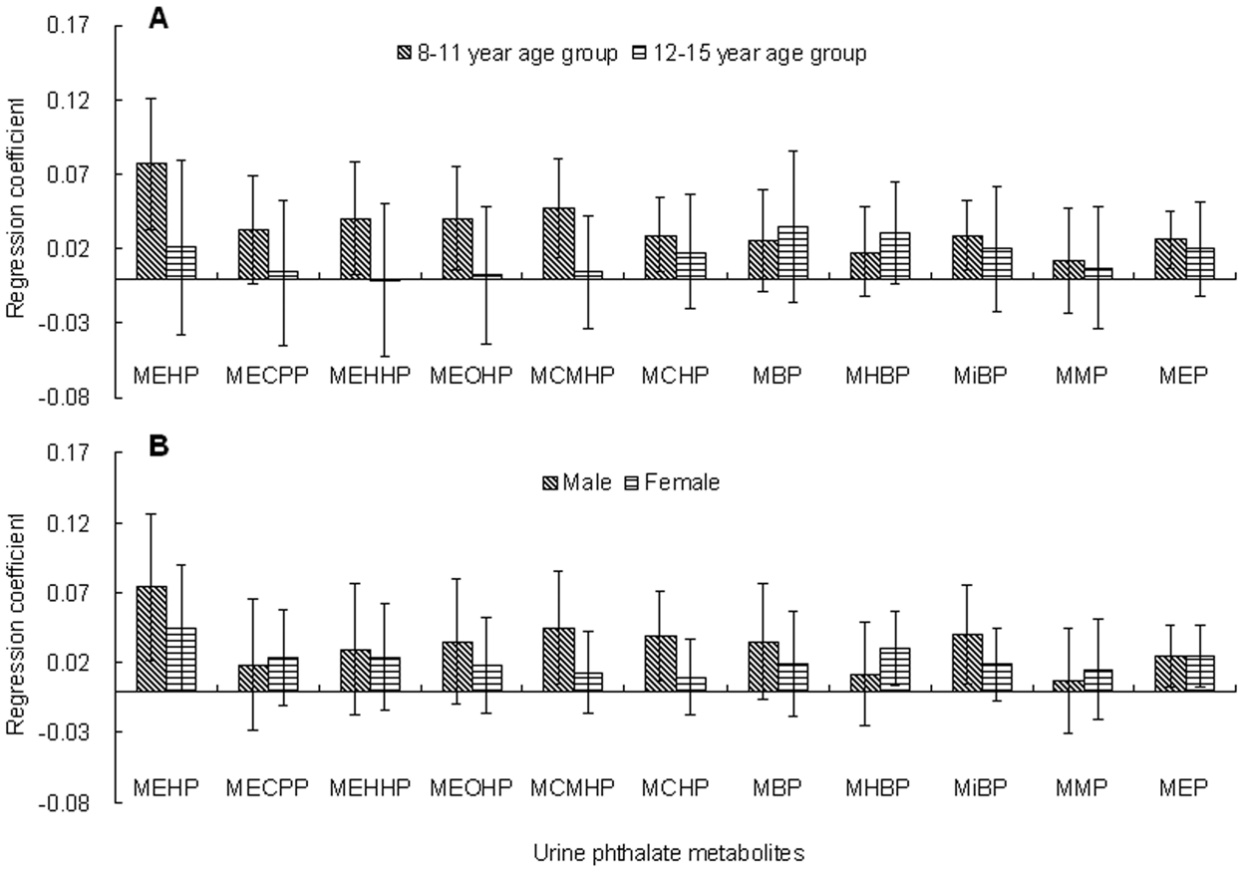
Comparison of the associations of urine phthalate metabolites corrected by specific gravity with body mass index between age subgroups (A) or sex (B) by multiple linear regression in 259 school children of Shanghai, China in 2012 (A: adjustment for age in years and sex; B: adjustment for age; regression coefficient: log-transformed BMI/log-transformed metabolite concentration.; Error bar: 95% confidence interval).

As shown in Figure 1, and Tables S6 and S7 in Supporting Information File S1, the associations between urine metabolites and BMI showed some sex- or age-specific differences in adjusted model 1. Most of these associations tended to be stronger in younger children than in older ones and in males than in females although that interaction test suggested that the associations of phthalate metabolites with BMI did not change by age or sex with an exception of MCMHP by age (p = 0.061). Interestingly, both MEHHP and MEOHP were found to be significantly associated with BMI in the 8–11 year age group (Table S6 in Supporting Information File S1), but these associations were not significant in all subjects (Table 4). After adjusted for additional urine phthalate metabolites in adjusted model 2, these significant associations disappeared (data not shown).

## Discussion

On the whole, urine phthalate metabolite concentrations in this study were comparable to those from previous studies conducted in other countries (Table S8 in Supporting Information File S1), although the profiles of urinary phthalate metabolites demonstrated some differences in terms of quantity and species of metabolites. For example, MBzP was detected only in 38.6% of subjects in this study, but detected in almost 100% in the studies of U.S., German, and Spanish children [32], [44], [45]. These differences may primarily reflect regional differences in use of phthalates. Additionally, different race and urine type (first morning urine in this study) may contribute to these differences as well.

As a result of an extensive use of phthalate-related products, human can be exposed to phthalates through almost all possible pathways, such as ingestion, inhalation, dermal contact, and placenta transmission [7]. Generally speaking, ingestion of food is the major exposure route of phthalates, in particular for high molecular weight phthalates, such as DEHP and BBzP [46]. For low molecular weight phthalates, such as DBP, DiBP, and DMP, inhalation of indoor air or dermal contact may be relevant exposure pathways next to food ingestion [47]. For DEP, one of low molecular weight phthalates, the major exposure route may largely be through dermal contact (especially by use of personal-care products) [48]. It was expected that exposure pathway-related variables, such as caloric intake and food composition related to food ingestion, and daily indoor time related to inhalation of indoor air, were positively associated with corresponding phthalate exposures, and then urine phthalate metabolites. Sum of DEHP and MCHP may be proxies for food ingestion-related variables, such as caloric intake and food composition, MMP may be for daily indoor time, and MEP may be for use of personal-care products, and sum of DBP or MiBP may be related to both food ingestion and daily indoor time.

In the present study we found nine urine phthalate metabolites and five sums were positively associated with BMI or WC in Chinese school children when adjusted for individual SG, age, and sex (in adjusted model 1), but most of these significant associations disappeared and some even reversed when further adjusted for other urine phthalate metabolites (in adjusted model 2). Only MEHP and MEP remained significant. Close correlations among phthalate metabolite measurements may be one reason for these association changes (Table S4 in Supporting Information File S1), and these changes may mean other possibilities. One possibility was that some phthalate metabolites might act as confounders. When these metabolites were included as covariables, they corrected the associations of metabolites of interest with BMI or WC. Another possibility was that some phthalates might have modifying effects on BMI or WC [49], [50]. A simple adjustment might diminish true associations of metabolites of interest with BMI or WC. So it was expected that a still significant association might be more reliable after adjustment for other urine phthalate metabolites.

Given the cross-sectional nature of this study, the possibility of reverse causation cannot be ruled out. It is possible that increased overall caloric intake, different diet composition, and longer daily indoor time for obese individuals might increase the risk of phthalate exposures, compared to normal weight ones [51]. These variables related to obesity were also confounders for the relationships between phthalate exposures and obesity. Lack of information on these obesity-related variables was an important limitation for this study. Fortunately, since urine phthalate metabolites could be proxies for these confounders as discussed above, the inclusion of urine phthalate metabolites as covariables in multiple linear regression model (adjusted model 2) corrected potential effects of these confounders unavailable in this study to some extent. Therefore, it should be more valid for the presence of significant associations of MEHP and MEP with BMI or WC after adjustment for age, sex as well as other urine phthalate metabolites.

An underlying assumption in this study and other similar studies is that one single measurement of spot urine could represent a long-term exposure of corresponding participant to no-persistent environmental chemicals [52]. Given short biologic half-lives of phthalate metabolites (12 hours or less), multiple sources and exposure routes of phthalates, varying diet composition and behavior of participants, urine collection time in a day and urine dilution, one single measurement of spot urine is not likely to perfectly represent a long-term exposure [53]. Some researches assessed the prediction of single spot urine for a long-term exposure and reported to have a moderate predictabilityapp:ds:power for long-term exposure and this ability differed by phthalate metabolites [53]. For example, Hauser et al. reported that single spot urine was moderately predictive of each subject’s exposure tertile categorization over three months with the sensitivities ranging from 0.56 to 0.74, and most predictive for MEP and least predictive for MEHP among five metabolites in 11 adult men [39]. Teitelbaum et al. assessed the variability of eight creatinine-corrected phthalate metabolites over six months and found moderate reproducibilities with intraclass correlation coefficients (ICCs) ranging from 0.21 (mono(3-carboxypropyl) phthalate, MCPP) to 0.62 (MBzP) in 35 children 6–10 years of age [54]. Additionally, misclassification due to one measurement of spot urine might result in an attenuated estimate of association of phthalate metabolite exposures with BMI or WC, which meant that significant associations based on spot urine were conservative [51]. In this study, we used first morning urine instead of spot one. Because the urine collection time is similar in a day for first morning urine, it decreases the variations of urine phthalate metabolite concentration. This strengthened the predictive ability of one measurement for long-term exposure than pure spot urine [53].

In our studies, we observed some age- or sex-related differences in the associations of phthalate metabolites with BMI or WC. MEHHP and MEOHP were found to be significantly associated with BMI only in the 8–11 year age group, not in all the subjects. These age- or sex-related differences were also reported in some previous studies [29], [30]. These differences might be related to different status of sex hormone endocrine of participants between sex or age subgroups (8–11 or 12–15 year age group). Puberty usually begins between 10 and 13 years of age, and serum levels of endogenous sex hormone in puberty significantly rise, especially for estradiol in girls and testosterone in boys. The differences in sex hormone function may lead to different susceptibility of participants to sex hormone perturbation caused by phthalate exposure [35]. More accurate measures of sex hormone levels and puberty status are needed to further explore the biologic mechanisms for the interrelationship among sex hormone, phthalate exposures and BMI.

There were some epidemiologic studies supporting the associations of postnatal phthalate exposures and body size measures in children. Using data from the National Health and Nutrition Examination Survey (NHANES) 1999–2002, Hatch et al. found that BMI increased with urinary MEP quartiles and inversely related to MEHP quartiles in girls 12–19 years of age, but no metabolite quartiles were significantly associated with BMI in the 6–11 year age group [29]. Boas et al. observed that some metabolites, such as MEP, MEOHP, MECPP, and sum of DEHP metabo­lites, negatively correlated with BMI in 845 Danish children 4–9 years of age although this study was primarily designed to investigate the association of urine phthalate metabolites and thyroid function, insulin-like factor 1, and growth [24]. Teitelbaum et al. reported no significant associations of urinary nine phthalate metabolites at baseline with BMI or WC measured one year later in 387 Hispanic and Black children 6–8 years of age in New York City; however, after divided into normal weight and overweight groups, the data showed significant associations of MEP and sum of low molecular-weight (MEP, MBP, and MiBP) with BMI and WC in overweight children [30]. Our study results were comparable to those from Teitelbaum et al. and Hatch et al., except for an inverse association of MEHP with BMI reported by Hatch et al., but distinctly different from those by Boas et al.

There are some plausible explanations for these discrepancies. Firstly, creatinine correction for urine phthalate metabolite concentrations can weaken or even reverse the association due to the positive associations of urine phthalate metabolite concentrations and BMI with urinary creatinine [30], [40]. In this circumference, SG correction may be a better choice [55]. The studies conducted by Teitelbaum et al. and Hatch et al. used creatinine to correct the urine dilution. Secondly, some studies including the study by Boas et al. reported that phthalates, such as DEHP and DiNP, might suppress thyroid hormone levels and insulin-like growth factor I (IGF-I) in serum [23], [24]. This suppression would decrease the growth of children, and therefore BMI values. When this trend exceeded the adipogenesis effect caused by phthalates, phthalate exposures would be negatively associated with BMI. Because younger children experience more rapid growth, their growth is more likely to be affected by external disturbance. This might explain the negative associations of phthalate exposures with BMI in the study of younger study population by Boas et al. [24]. There are some mice studies that have shown negative associations between prenatal phthalate exposures, such as DBP, BBP, and DEHP, and offspring weight [56], [57]. If these explanations are reasonable, DEP, DEHP, DBP, and DiBP are main phthalates associated with BMI or WC.

### Conclusions

In this study we found nine urinary phthalate metabolites and three sums were positively associated with BMI or WC in Chinese school children, and it was more evident for MEHP and MEP. Some associations showed sex and/or age related difference. Given the cross-sectional nature of this study and lack of some important obesity-related variables, the associations discovered here need to be further investigated.

## Supporting Information

Supporting Information File S1 **Table S1** BMI-based criteria by age and sex for screening of overweight and obese school children proposed by Working Group on Obesity in China (WGOC) in 2005. **Table S2** Settings for multiple reaction monitoring (MRM) mode. **Table S3** Results of quality assurance/quality control. **Table S4** Pearson correlation coefficients among phthalate metabolites uncorrected by specific gravity. **Table S5** Calculation of sampling weights of each selected school (sampling weights = number of source population/number of participants). **Table S6** Association of urinary phthalate metabolite concentrations corrected by specific gravity with body mass index by age groups after adjustment for age in years and sex in 259 school children of Shanghai, China in 2011–2012. **Table S7** Association of urinary phthalate metabolite concentrations corrected by specific gravity with body mass index by sex after adjustment for age in 259 school children of Shanghai, China in 2011–2012. **Table S8** Comparison of urinary phthalate metabolite concentrations (geometric mean, ng/mL) (detection frequency, %) between this study and other several populations.
(DOC)
