# Supplementary material for: Urinary Phthalate Metabolites Are Associated with Body Mass Index and Waist Circumference in Chinese School Children
Source: PLoS One. 2013 Feb 20;8(2):e56800. doi: 10.1371/journal.pone.0056800 (PMC3577690; doi:10.1371/journal.pone.0056800)
Supplement: Supporting Information File S1 — Table S1 BMI-based criteria by age and sex for screening of overweight and obese school children proposed by Working Group on Obesity in China (WGOC) in 2005. Table S2 Settings for multiple reaction monitoring (MRM) mode. Table S3 Results of quality assurance/quality control. Table S4 Pearson correlation coefficients among phthalate metabolites uncorrected by specific gravity. Table S5 Calculation of sampling weights of each selected school (sampling weights = number of source population/number of participants). Table S6 Association of urinary phthalate metabolite concentrations corrected by specific gravity with body mass index by age groups after adjustment for age in years and sex in 259 school children of Shanghai, China in 2011–2012. Table S7 Association of urinary phthalate metabolite concentrations corrected by specific gravity with body mass index by sex after adjustment for age in 259 school children of Shanghai, China in 2011–2012. Table S8 Comparison of urinary phthalate metabolite concentrations (geometric mean, ng/mL) (detection frequency, %) between this study and other several populations. (DOC) [file pone.0056800.s001.doc]

**Supporting Information**

**Urinary Phthalate Metabolites are Associated with Body Mass Index and waist circumference in Chinese School Children**

Hexing Wang,^1^ Ying Zhou, ^1*^ Chuanxi Tang, ^2^ Jingui Wu,^2^ Yue Chen, ^3^ Qingwu Jiang^1^

^1^ Key Laboratory of Public Health Safety of Ministry of Education, School of Public Health, Fudan University，Shanghai, 200032, China

^2^ Centers for Disease Control and Prevention of Changning District, Shanghai, 200051, China

^3^ Department of Epidemiology and Community Medicine, Faculty of Medicine, University of Ottawa, Ottawa, Ontario, Canada

**Chemicals and Reagents.**

Thirteen phthalate metabolites, including mono(2-ethylhexyl) phthalate (MEHP), mono(2-ethyl-5-carboxypentyl) phthalate (MECPP), mono[(2-carboxymethyl) hexyl] phthalate (MCMHP), mono(2-ethyl-5-oxohexyl) phthalate (MEOHP) and mono(2-ethyl-5-hydroxyhexyl) phthalate (MEHHP), mono-n-butyl phthalate (MBP), monoisobutyl phthalate (MiBP), monomethyl phthalate (MMP), monoethyl phthalate(MEP), monocyclohexyl phthalate (MCHP), monobenzyl phthalate (MBzP), monoisonoyl phthalate (MiNP), and monooctyl phthalate (MOP), and 4-thylumbelliferone, and seven isotopically labeled internal standards, incliding 13C_4_-MMP, 13C_4_-MEP, 13C_4_-MBP, 13C_4_-MECPP, 13C_4_-MEHP, 13C_4_-MBzP, and 13C_4_-4-methylumbelliferone, were obtained from Cambridge Isotope Laboratories (MA, USA).

Mono (4-hydroxybutyl) phthalate (MHBP, ＞98 in purity), 4-methylumbelliferone glucuronide (＞97 in purity), β-glucuronidase/sulfatase from Helix pomatia (Type H-2，[≥100,000 Sigma units /mL](http://www.sigmaaldrich.com/catalog/product/sigma/g7017?lang=zh&region=CN) ), acetic acid of LC-MS grade, and formic acid of LC-MS grade were purchased from Sigma-Aldrich (Shanghai, China). Three reagents of LC-MS grade, including water, methanol, and acetonitrile, were obtained from Fisher (Fair Lawn, NJ, USA). Three reagents of analytical grade, including acetic aid, sodium bicarbonate, and ammonium acetate, were purchased from Guoyao (Shanghai, China) and used for sample pretreatment. Deionized water was from Milli-Q-Plus (Millipore, Bedford, MA, USA) and used for sample pretreatment.

**Sample Preparation**

An aliquot (1.0 mL) of urine was spiked with 50 μL of stand solution containing 500 ng/mL of six mixed isotopically labeled phthalate metabolites, 500ng/mL of 13C_4_-4-methylumbelliferone, and 1000 ng/mL of 4-methylumbelliferone glucuronide, and vortexed for 30s. After 30min of equilibration, the sample was buffered with 200 μL of 1.0 M ammonium acetate buffer (pH 5.0; 7.7 g of ammonium acetate dissolved in 100 mL of Milli-Q water and added 6 mL of acetic acid) and added by 15μL of β-glucuronidase/sulfatase, followed by incubation for overnight at 37℃.

After enzymatic deconjugation, the urine samples were loaded onto Oasis MAX solid-phase extraction (SPE) anion exchange cartridges (150mg/mL, Waters, Milford, MA). The cartridges were previously preconditioned with 4 mL of methanol and 4 mL of Milli-Q water. Ammonium acetate buffer was washed from the cartridges by 4 mL of pure water, and 4 ml of 50 mM sodium bicarbonate buffer (pH 8.5; 2.1 g of sodium bicarbonate dissolved in 500 mL of Milli-Q water) was used to promote the binding of phthalate metabolites to SPE material. After neutral or basic compounds were washed from the cartridges by 4 mL of methanol, the SPE material was under vacuum for 10 min. The target phthalate metabolites were eluted by 5 mL of methanol containing 2% formic acid (v/v). Flow rate of loading, washing, and elution in SPE process was kept at 1.5 mL/min. The eluate was concentrated to dryness with a gentle stream of nitrogen in a 45℃ water bath, and reconstituted in 0.5mL of 30% acetonitrile aqueous solution (v/v). The final solution was transferred into a 2mL amber glass vial and kept at 4℃ until analysis.

**Instrumental Analysis**

A Waters Acquity ultra-performance liquid chromatography (UPLC) coupled with a Waters Xevo TQ triple quadrupole mass spectrometer (Milford, MA, USA) was used for the measurement of target compounds. Ten microliters of final solution were injected onto an Acquity UPLC HSS T3 column (100mm×2.1mm×1.8µm). Target compounds were eluted by water (A) and acetonitrile (B) both containing 0.1% acetic acid (v/v) at a flow rate of 300µL/min. The gradient of organic modifier (B) varied linearly as follows: 0-0.2min, 5%B; 0.2-1.0min, 25%B; 1.0-9.0min, 55%B; 9.0-11.0min, 100%B; 11.0-11.5min, 5%B; 11.5-14.0min, 5%B. The column temperature was set to 40℃.

13C_4_-MMP was used as a surrogate standard for quantification of MHBP and MMP, 13C_4_-MEP for MEP, 13C_4_-4-methylumbelliferone for 4-methylumbelliferone, 13C_4_-MECPP for MECPP and MEHHP, 13C_4_-MBP for MBP, MiBP, MEOHP, and MCMHP, 13C_4_-MBzP for MBzP and MCHP, 13C_4_-MEHP for MEHP, MOP, and MiNP.

Target compounds were analyzed under negative ion (NI) mode. Nitrogen was used as the desolvation gas. The desolvation gas flow was set to 800L/h and its temperature was set to 400℃. The cone gas flow was set to 40 L/h, and the source temperature was set to 120℃. The capillary voltage was set to 2.8 KV. The multiple reaction monitoring (MRM) mode was used, and the specific setting was listed in Table S2.

**Quality Assurance/Quality Control**

For each batch of 20 samples analyzed, two procedural blanks and a pair of matrix spiked samples at 50 ng/mL were processed. An internal standard calibration curve of six concentration levels, ranging from 0.2 ng/mL to 100 ng/mL, was prepared every batch. As a check for instrumental drift in response factors and carry-over of phthalate metabolites from sample to sample, a midpoint calibration standard and a procedural blank were injected every 10 samples. The correlation coefficient of calibration curves was above 0.995. As shown in Table S3, the recoveries of target compounds in matrix spiked samples at 50 ng/mL varied between 70.3% and 124%, and their relative standard deviation varied between 9.2% and 19.1%. MEHP, MiBP, MBP, MMP, and MEP were detected in procedural blanks, and sample concentrations for these compounds were subtracted with corresponding blank values. The limits of quantitation (LOQ) and limits of detection (LOD) for target compounds were 0.10-0.80 ng/mL and 0.03 -0.24 ng/mL, respectively. The recoveries of 4-methylumbelliferone glucuronide spiked in all samples were between 92% and 109%, which indicated that phthalate metabolite glucuronide was completely deconjugated by β-glucuronidase/sulfatase.

Table S1. BMI-based criteria by age and sex for screening of overweight and obese school children proposed by Working Group on Obesity in China (WGOC) in 2005.

| Age(years) | Boys | | | Girls | | |
| --- | --- | --- | --- | --- | --- | --- |
|  | Normal | Overweight | Obesity | Normal | Overweight | Obesity |
| 7- | 13.5-17.3 | 17.4-19.1 | ≥19.2 | 13.4-17.1 | 17.2-18.8 | ≥18.9 |
| 8- | 13.7-18.0 | 18.1-20.2 | ≥20.3 | 13.5-18.0 | 18.1-19.8 | ≥19.9 |
| 9- | 13.9-18.8 | 18.9-21.3 | ≥21.4 | 13.6-18.9 | 19.0-20.9 | ≥21.0 |
| 10- | 14.2-19.5 | 19.6-22.4 | ≥22.5 | 13.9-19.9 | 20.0-22.0 | ≥22.1 |
| 11- | 14.5-20.2 | 20.3-23.5 | ≥23.6 | 14.3-21.0 | 21.1-23.2 | ≥23.3 |
| 12- | 14.9-20.9 | 21.0-24.6 | ≥24.7 | 14.8-21.8 | 21.9-24.4 | ≥24.5 |
| 13- | 15.5-21.8 | 21.9-25.6 | ≥25.7 | 15.7-22.5 | 22.6-25.5 | ≥25.6 |
| 14- | 16.2-22.5 | 22.6-26.3 | ≥26.4 | 16.4-22.9 | 23.0-26.2 | ≥26.3 |
| 15- | 16.8-23.0 | 23.1-26.8 | ≥26.9 | 17.0-23.3 | 23.4-26.9 | ≥26.9 |
| 16- | 17.3-23.4 | 23.5-27.3 | ≥27.4 | 17.5-23.6 | 23.7-27.3 | ≥27.4 |
| 17- | 17.7-23.7 | 23.8-27.7 | ≥27.8 | 17.7-23.7 | 23.8-27.6 | ≥27.7 |
| 18 | 18.1-23.9 | 24.0-27.9 | ≥28.0 | 17.9-23.9 | 24.0-27.9 | ≥28.0 |

Table S2. Settings for multiple reaction monitoring (MRM) mode.

| Compounds | Sampling cone voltage (V) | Collision energy (V) | Precursor ion | Product ion |
| --- | --- | --- | --- | --- |
| MEHP | 25 | 19 | 277 | 134 |
| MECPP | 15 | 20 | 307 | 159 |
| MEHHP | 25 | 26 | 293 | 121 |
| MEOHP | 25 | 21 | 291 | 121 |
| MCMHP | 10 | 20 | 307 | 159 |
| MiBP | 20 | 24 | 221 | 77 |
| MBP | 20 | 21 | 221 | 77 |
| MMP | 15 | 23 | 179 | 77 |
| MEP | 20 | 25 | 193 | 77 |
| MCHP | 25 | 25 | 247 | 77 |
| MHBP | 20 | 17 | 237 | 121 |
| MBzP | 20 | 17 | 255 | 183 |
| MiNP | 30 | 20 | 291 | 121 |
| MOP | 25 | 22 | 277 | 125 |
| 4-methylumbelliferone | 20 | 34 | 175 | 119 |

Table S3. Results of quality assurance/quality control.

| Compounds | Procedural blank (ng/mL) | Recovery of spiked Matrix (%) | Relative standard deviations (%) | LOD (ng/mL) | LOQ (ng/mL) |
| --- | --- | --- | --- | --- | --- |
| MEHP | 4.6 | 84.2-124 | 19.1 | 0.03 | 0.10 |
| MECPP | ＜LOD | 82.6-113 | 15.5 | 0.16 | 0.53 |
| MEHHP | ＜LOD | 86.5-108 | 11.1 | 0.11 | 0.37 |
| MEOHP | ＜LOD | 76.8-92.3 | 9.2 | 0.10 | 0.33 |
| MCMHP | ＜LOD | 80.6-103 | 12.2 | 0.06 | 0.20 |
| MiBP | 1.3 | 72.2-94.3 | 13.3 | 0.09 | 0.30 |
| MBP | 1.1 | 92.3-117 | 11.8 | 0.10 | 0.33 |
| MMP | 1.5 | 76.4-98.6 | 12.7 | 0.12 | 0.40 |
| MEP | 1.2 | 70.3-98.0 | 16.5 | 0.13 | 0.43 |
| MCHP | ＜LOD | 86.9-118 | 15.2 | 0.05 | 0.17 |
| MHBP | ＜LOD | 72.3-104 | 18.0 | 0.24 | 0.80 |
| MBzP | ＜LOD | 83.2-106 | 12.1 | 0.04 | 0.13 |
| MiNP | ＜LOD | 95.6-121 | 11.7 | 0.03 | 0.10 |
| MOP | ＜LOD | 88.6-117 | 13.8 | 0.03 | 0.10 |

Table S4. Pearson correlation coefficients among phthalate metabolites uncorrected by specific gravity.

| Compounds | MEHP | MECPP | MEHHP | MEOHP | MCMHP | Sum of DEHP | MCHP | Sum of high MWP | MBP | MHBP | Sum of DBP | MiBP | MMP | MEP | Sum of low MWP | Sum of all |
| --- | --- | --- | --- | --- | --- | --- | --- | --- | --- | --- | --- | --- | --- | --- | --- | --- |
| MEHP | 1 | 0.598 | 0.533 | 0.623 | 0.524 | 0.747 | 0.361 | 0.748 | 0.431 | 0.3400 | 0.494 | 0.418 | .493 | 0.332 | 0.497 | 0.613 |
| MECPP |  | 1 | 0.920 | 0.951 | 0.859 | 0.948 | 0.314 | 0.946 | 0.518 | 0.441 | 0.565 | 0.462 | .489 | 0.334 | 0.570 | 0.668 |
| MEHHP |  |  | 1 | 0.929 | 0.812 | 0.908 | 0.292 | 0.905 | 0.461 | 0.4000 | 0.513 | 0.422 | .398 | 0.287 | 0.507 | 0.609 |
| MEOHP |  |  |  | 1 | 0.888 | 0.959 | 0.330 | 0.957 | 0.559 | 0.475 | 0.632 | 0.522 | .523 | 0.342 | 0.632 | 0.701 |
| MCMHP |  |  |  |  | 1 | 0.869 | 0.313 | 0.868 | 0.583 | 0.499 | 0.649 | 0.537 | .565 | 0.350 | 0.653 | 0.710 |
| Sum of DEHP |  |  |  |  |  | 1 | 0.354 | 0.962 | 0.551 | 0.448 | 0.605 | 0.502 | .532 | 0.359 | 0.613 | 0.729 |
| MCHP |  |  |  |  |  |  | 1 | 0.364 | 0.301 | 0.294 | 0.366 | 0.295 | .326 | 0.214 | 0.365 | 0.383 |
| Sum of high MWP |  |  |  |  |  |  |  | 1 | 0.557 | 0.475 | 0.605 | 0.499 | .534 | 0.353 | 0.612 | 0.739 |
| MBP |  |  |  |  |  |  |  |  | 1 | 0.718 | 0.597 | 0.276 | .488 | 0.340 | 0.688 | 0.653 |
| MHBP |  |  |  |  |  |  |  |  |  | 1 | 0.618 | 0.259 | .520 | 0.304 | 0.628 | 0.597 |
| Sum of DBP |  |  |  |  |  |  |  |  |  |  | 1 | 0.820 | .610 | 0.389 | 0.905 | 0.802 |
| MiBP |  |  |  |  |  |  |  |  |  |  |  | 1 | .465 | 0.331 | 0.690 | 0.627 |
| MMP |  |  |  |  |  |  |  |  |  |  |  |  | 1 | 0.385 | 0.666 | 0.623 |
| MEP |  |  |  |  |  |  |  |  |  |  |  |  |  | 1 | .0637 | 0.559 |
| Sum of low MWP |  |  |  |  |  |  |  |  |  |  |  |  |  |  | 1 | 0.873 |
| Sum of all |  |  |  |  |  |  |  |  |  |  |  |  |  |  |  | 1 |

All coefficients are significant at p＜0.01.

Table S5 Calculation of sampling weights of each selected school (sampling weights=number of source population/number of participants).

| School |  | Total | Normal weight | Over weight | Obesity | Low weight |
| --- | --- | --- | --- | --- | --- | --- |
| Priamry 1 | Source population | 448 | 239 | 56 | 89 | 64 |
|  | Participants |  | 19 | 8 | 16 |  |
|  | Sampling weights |  | 12.58 | 7.00 | 5.56 |  |
| Priamry 2 | Source population | 518 | 262 | 72 | 120 | 64 |
|  | Participants |  | 22 | 7 | 15 |  |
|  | Sampling weights |  | 11.91 | 10.29 | 8.00 |  |
| Priamry 3 | Source population | 573 | 277 | 84 | 96 | 116 |
|  | Participants |  | 21 | 8 | 12 |  |
|  | Sampling weights |  | 13.19 | 10.50 | 8.00 |  |
| Middle 1 | Source population | 374 | 162 | 36 | 83 | 93 |
|  | Participants |  | 18 | 9 | 14 |  |
|  | Sampling weights |  | 9.00 | 4.00 | 5.93 |  |
| Middle 2 | Source population | 467 | 222 | 62 | 78 | 105 |
|  | Participants |  | 20 | 10 | 13 |  |
|  | Sampling weights |  | 11.10 | 6.20 | 6.00 |  |
| Middle 3 | Source population | 541 | 243 | 78 | 99 | 121 |
|  | Participants |  | 24 | 11 | 12 |  |
|  | Sampling weights |  | 10.13 | 7.09 | 8.25 |  |

Table S6. Association of urinary phthalate metabolite concentrations corrected by specific gravity with body mass index by age groups after adjustment for age in years and sex in 259 school children of Shanghai, China in 2011-2012.

| Compounds | 8-11 year age group | | 12-15 year age group | | Interaction |
| --- | --- | --- | --- | --- | --- |
|  | β (95% CI) | p-Value | β (95% CI) | p-Value | p-Value |
| MEHP | 0.077(0.034,0.121) | 0.001 | 0.021(-0.037,0.079) | 0.471 | 0.162 |
| MECPP | 0.033(-0.003,0.069) | 0.073 | 0.004(-0.044,0.053) | 0.868 | 0.228 |
| MEHHP | 0.040(0.003,0.078) | 0.035 | -0.001(-0.052,0.051) | 0.978 | 0.145 |
| MEOHP | 0.040(0.006,0.075) | 0.023 | 0.002(-0.044,0.048) | 0.936 | 0.135 |
| MCMHP | 0.047(0.014,0.080) | 0.006 | 0.004(-0.034,0.042) | 0.830 | 0.061 |
| Sum of DEHP | 0.054(0.015,0.093) | 0.008 | 0.011(-0.043,0.064) | 0.699 | 0.145 |
| MCHP | 0.029(0.003,0.054) | 0.027 | 0.018(-0.020,0.056) | 0.347 | 0.744 |
| Sum of high MWP | 0.054(0.015,0.093) | 0.007 | 0.017 (-0.038,0.071) | 0.546 | 0.189 |
| MBP | 0.025(-0.008,0.059) | 0.139 | 0.035(-0.016,0.086) | 0.175 | 0.757 |
| MHBP | 0.018(-0.012,0.048) | 0.232 | 0.031(-0.003,0.065) | 0.071 | 0.565 |
| Sum of DBP | 0.033(0.001,0.066) | 0.043 | 0.037(-0.012,0.085) | 0.135 | 0.844 |
| MiBP | 0.029(0.004,0.053) | 0.023 | 0.020(-0.023,0.062) | 0.360 | 0.759 |
| MMP | 0.012(-0.022,0.047) | 0.483 | 0.007(-0.034,0.048) | 0.736 | 0.886 |
| MEP | 0.026(0.007,0.045) | 0.007 | 0.020(-0.011,0.052) | 0.195 | 0.832 |
| Sum of low MWP | 0.038(0.004,0.073) | 0.031 | 0.043(-0.009,0.095) | 0.103 | 0.750 |
| Sum of all | 0.047(0.008,0.085) | 0.017 | 0.047(-0.012,0.105) | 0.117 | 0.992 |

β: Regression coefficient(log-transformed BMI /log-transformed metabolite concentration).

Table S7. Association of urinary phthalate metabolite concentrations corrected by specific gravity with body mass index by sex after adjustment for age in 259 school children of Shanghai, China in 2011-2012.

| Compounds | Male | | Female | | Interaction |
| --- | --- | --- | --- | --- | --- |
|  | β (95% CI) | p-Value | β (95% CI) | p-Value | p-Value |
| MEHP | 0.074(0.022,0.126) | 0.005 | 0.045(0.001,0.089) | 0.045 | 0.499 |
| MECPP | 0.018(-0.029,0.065) | 0.447 | 0.024(-0.010,0.058) | 0.167 | 0.841 |
| MEHHP | 0.029(-0.018,0.076) | 0.229 | 0.024(-0.014,0.062) | 0.209 | 0.843 |
| MEOHP | 0.035(-0.008,0.079) | 0.112 | 0.018(-0.016,0.052) | 0.305 | 0.521 |
| MCMHP | 0.045(0.005,0.085) | 0.027 | 0.013(-0.017,0.042) | 0.408 | 0.212 |
| Sum of DEHP | 0.046(-0.003,0.095) | 0.067 | 0.030(-0.008,0.069) | 0.120 | 0.638 |
| MCHP | 0.039(0.007,0.071) | 0.017 | 0.010(-0.017,0.037) | 0.467 | 0.326 |
| Sum of high MWP | 0.049(0.001,0.099) | 0.051 | 0.031(-0.007,0.070) | 0.111 | 0.580 |
| MBP | 0.035(-0.006,0.076) | 0.091 | 0.019(-0.018,0.057) | 0.303 | 0.582 |
| MHBP | 0.012(-0.026,0.049) | 0.536 | 0.030(0.003,0.056) | 0.028 | 0.405 |
| Sum of DBP | 0.035(-0.004,0.075) | 0.078 | 0.036(0.000,0.071) | 0.050 | 0. 928 |
| MiBP | 0.040(0.006,0.075) | 0.023 | 0.019(-0.007,0.045) | 0.153 | 0.372 |
| MMP | 0.007(-0.031,0.045) | 0.731 | 0.015(-0.020,0.051) | 0.401 | 0.660 |
| MEP | 0.025(0.003,0.047) | 0.028 | 0.025(0.003,0.047) | 0.026 | 0.910 |
| Sum of low MWP | 0.042(-0.002,0.085) | 0.061 | 0.040(0.004,0.077) | 0.031 | 0.956 |
| Sum of all | 0.051(0.001,0.100) | 0.044 | 0.043(0.003,0.083) | 0.034 | 0.856 |

β: Regression coefficient(log-transformed BMI /log-transformed metabolite concentration).

Table S8. Comparison of urinary phthalate metabolite concentrations (geometric mean, ng/mL) (detection frequency, %) between this study and other several populations.

|  | U.S. [1] | U.S. [1] | German [2] | Spanish [3] | This study |
| --- | --- | --- | --- | --- | --- |
| Sample size | 327 | 682 | 599 | 30 | 259 |
| Year | 2007-2008 | 2007-2008 | 2003-2006 | 2005-2006 | 2011-2012 |
| Study population | Children 6-11 yeas | Children 12-19 yeas | children 3-14 years | children 4 years | children 8-15 years |
| MEHP | 2.39 (＞50) | 2.99 (＞50) | 6.4 (99.8) | Median 6.2 (100) | 21.3 (100) |
| MECPP | 46.6 (＞50) | 44.1 (＞50) | 62.5 (100) | Median 115 (100) | 28.8 (100) |
| MEHHP | 28.6 (＞50) | 29.8 (＞50) | 47.9 (100) | Median 57.4 (100) | 16.1 (100) |
| MEOHP | 16.9 (＞50) | 16.9 (＞50) | 37.0 (100) | Median 44.6 (100) | 22.9 (100) |
| MCMHP | - | - | 20.8 (100) | - | 22.0 (99.6) |
| MiBP | 10.7 (＞50) | 10.2 (＞50) | 94.3 (100) | Median 41.9 (100) | 38.9 (100) |
| MBP | 26.9 (＞50) | 26.2 (＞50) | 95.6 (100) | Median 30.2 (100) | 47.5 (99.6) |
| MMP | Median 1.20 (＞50) | 75% percentile 2.30 (＜50) | - | - | 9.6 (100) |
| MEP | 49.0 (＞50) | 98.6 (＞50) | - | Median 755 (100) | 15.3 (98.9) |
| MCHP | 0 (0) | 0 (0) | - | - | 0.80 (94.6) |
| MHBP | - | - | - | - | 26.6 (99.6) |
| MBzP | 15.4 (＞50) | 11.6(＞50) | 17.5 (100) | Median 33 (100) | Median 0.23 (38.6) |
| MOP | 0 (0) | 0 (0) | - | - | 0 (0) |
| MiNP | 90% percentile 1.39 (＜25) | 90% percentile 2.46 (＜25) | - | - | 0 (0) |

-, Not reported.

**References:**

1. CDC (Centers for Disease Control and Prevention) (2012) Fourth national report on human exposure to environmental ehemicals. Available: http://www.cdc.gov/exposurereport/pdf/FourthReport_UpdatedTables_Feb2012.pdf. Accessed 2012 july 1.

2. Becker K, Goen T, Seiwert M, Conrad A, Pick-Fuss H, et al. (2009) GerES IV: phthalate metabolites and bisphenol A in urine of German children. Int J Hyg Environ Health 212: 685-692.

3. Casas L, Fernandez MF, Llop S, Guxens M, Ballester F, et al. (2011) Urinary concentrations of phthalates and phenols in a population of Spanish pregnant women and children. Environ Int 37: 858-866.
